# Biomarkers of Systemic Inflammation and Growth in Early Infancy are Associated with Stunting in Young Tanzanian Children

**DOI:** 10.3390/nu10091158

**Published:** 2018-08-24

**Authors:** Sana Syed, Karim P. Manji, Christine M. McDonald, Rodrick Kisenge, Said Aboud, Christopher Sudfeld, Lindsey Locks, Enju Liu, Wafaie W. Fawzi, Christopher P. Duggan

**Affiliations:** 1 Division of Gastroenterology, Hepatology and Nutrition, Boston Children’s Hospital, Boston, MA 02115, USA; syedsana@gmail.com (S.S.); cmcdonald0407@gmail.com (C.M.M.); enju.liu@childrens.harvard.edu (E.L.); 2 Department of Pediatrics and Child Health, Muhimbili University of Health and Allied Sciences, Dar es Salaam, Tanzania; kpmanji@gmail.com (K.P.M.); rkisenge@muhas.ac.tz (R.K.); 3 Department of Microbiology and Immunology, Muhimbili University of Health and Allied Sciences, Dar es Salaam, Tanzania; aboudsaid@yahoo.com; 4 Department of Global Health and Population, Harvard T.H. Chan School of Public Health, Boston, MA 02115, USA; csudfeld@hsph.harvard.edu (C.S.); mina@hsph.harvard.edu (W.W.F.); 5 Department of Nutrition, Harvard T.H. Chan School of Public Health, Boston, MA 02115, USA; lml395@mail.harvard.edu; 6 Department of Epidemiology, Harvard T.H. Chan School of Public Health, Boston, MA 02115, USA

**Keywords:** biomarkers, stunting, inflammation, growth, Tanzania, children

## Abstract

Stunting can afflict up to one-third of children in resource-constrained countries. We hypothesized that low-grade systemic inflammation (defined as elevations in serum C-reactive protein or alpha-1-acid glycoprotein) in infancy suppresses the growth hormone–insulin-like growth factor (IGF) axis and is associated with subsequent stunting. Blood samples of 590 children from periurban Dar es Salaam, Tanzania, were obtained at 6 weeks and 6 months of age as part of a randomized controlled trial. Primary outcomes were stunting, underweight, and wasting (defined as length-for-age, weight-for-age and weight-for-length *z*-scores < −2) between randomization and endline (18 months after randomization). Cox proportional hazards models were constructed to estimate hazard ratios (HRs) and corresponding 95% confidence intervals (CIs) of time to first stunting, underweight, and wasting as outcomes, with measures of systemic inflammation, insulin-like growth factor-1 (IGF-1) and insulin-like growth factor binding protein-3 (IGFBP-3) as exposures, adjusting for numerous demographic and clinical variables. The incidences of subsequent stunting, underweight, and wasting were 26%, 20%, and 18%, respectively. In multivariate analyses, systemic inflammation at 6 weeks of age was significantly associated with stunting (HR: 2.14, 95% CI: 1.23, 3.72; *p* = 0.002). Children with higher levels of IGF-1 at 6 weeks were less likely to become stunted (HR: 0.58, 95% CI: 0.37, 0.93; *p* for trend = 0.019); a similar trend was noted in children with higher levels of IGF-1 at 6 months of age (HR: 0.50, 95% CI: 0.22, 1.12; *p* for trend = 0.07). Systemic inflammation occurs as early as 6 weeks of age and is associated with the risk of future stunting among Tanzanian children.

## 1. Introduction

Globally it is estimated that 156 million children under the age of 5 years were stunted in 2015 [1]. Stunting is associated with increased risk of diarrhea, pneumonia, reduced neurocognitive capability, and diminished immunocompetence and accounts for about 1.2 million deaths annually in children under 5 years of age [2,3]. Furthermore, stunting is associated with oral vaccine failure; seroconversion rates for polio vaccination have been reported to be substantially lower among stunted versus nonstunted children [4]. In resource—constrained settings, dramatic linear growth faltering often occurs during the first 24 months of life [5]. This suggests that ameliorating growth faltering early in infancy may be particularly advantageous in reducing mortality, improving future cognition, and improving vaccine response [2,3,4].

Clinical studies in children with chronic inflammation, including inflammatory bowel disease and chronic kidney disease, have shown that increased concentrations of inflammatory biomarkers and reciprocally decreased concentrations of anabolic growth factors such as insulin-like growth factor-1 (IGF-1) are associated with linear growth faltering [6,7,8,9]. In resource-constrained settings, studies have shown that recurrent enteric and/or respiratory infections are associated with reduced growth velocity and stunting in children [10,11,12]. Recent data from Brazil and Zimbabwe have identified relationships between infection, inflammation (as measured by high-sensitivity C-reactive protein (hsCRP), and growth hormone (GH) resistance, whereas increased levels of GH, IGF-1, and IGF binding protein-3 (IGFBP-3) were associated with growth indices independent of hsCRP [13,14]. Investigators from Bangladesh and Brazil have also reported that enteric inflammation, measured by elevated fecal myeloperoxidase or alpha-1-antitrypsin, was associated with subsequent decreases in linear growth [15,16]. However, with the exception of the Bangladesh site, the remaining studies above were limited in their ability to assess causal relationships given their case-control design.

The present study was designed to prospectively assess the contribution of inflammation and anabolic growth markers during early infancy to subsequent early childhood stunting. We aimed to determine the prospective association of inflammatory (alpha-1-acid glycoprotein (AGP), C-reactive protein (CRP)) and growth (insulin-like growth factor 1 (IGF-1) and principal binding protein of circulating IGF-1 concentration (IGFBP3)) biomarkers with stunting, wasting, and underweight outcomes in a prospective cohort of young Tanzanian children at risk for growth faltering.

## 2. Materials and Methods

### 2.1. Study Design and Participants

Subjects were part of a randomized, double-blind, placebo-controlled trial designed to investigate whether daily administration of zinc and/or multivitamins (vitamin C, vitamin E, thiamin, riboflavin, niacin, vitamin B6, folate, and vitamin B12) to Tanzanian children would reduce the risk of infectious morbidity and improve growth compared with placebo. Results of this trial have been published previously [17]. Briefly, HIV-uninfected mothers of potentially eligible children were recruited into the study and their infants were randomized to 1 of the 4 study arms between 5 and 7 weeks of age. Children of multiple births and those with congenital anomalies or other conditions that would interfere with the study procedures were excluded from the trial. Subjects were followed for 18 months.

Birth characteristics were obtained immediately after delivery whenever possible. At the time of randomization, a study physician performed a clinical examination and drew a blood sample, and a study nurse performed a history of morbidity and infant feeding practices and anthropometric measurements. Mothers were asked to return to the study clinic with their children every 4 weeks for data collection and standard clinical care, including growth monitoring, immunizations, routine medical treatment for illnesses, and periodic vitamin A supplementation (100,000 IU at 9 months and 200,000 IU at 15 months). At these visits, study nurses performed a morbidity history based on maternal recall aided by the mother’s symptom diary that she received at the previous visit. The symptom diary was a pictorial aid of illness symptoms (e.g., diarrhea, vomiting) where mothers were asked to check off which days their child had experienced these symptoms. The study nurses also measured the children’s weight using a digital infant balance with 10 g precision (Tanita) and their length with 1 mm precision using a rigid length board with a movable foot piece. Length-for-age *z*-scores (LAZ), weight-for-length *z*-scores (WLZ), and weight-for-age *z*-scores (WAZ) were calculated by using World Health Organization (WHO) child growth standards [18]. Stunting, wasting, and underweight were defined as a LAZ, WLZ, and WAZ of 2 or more standard deviations (SDs) below the WHO population median, respectively.

**Figure 1:**
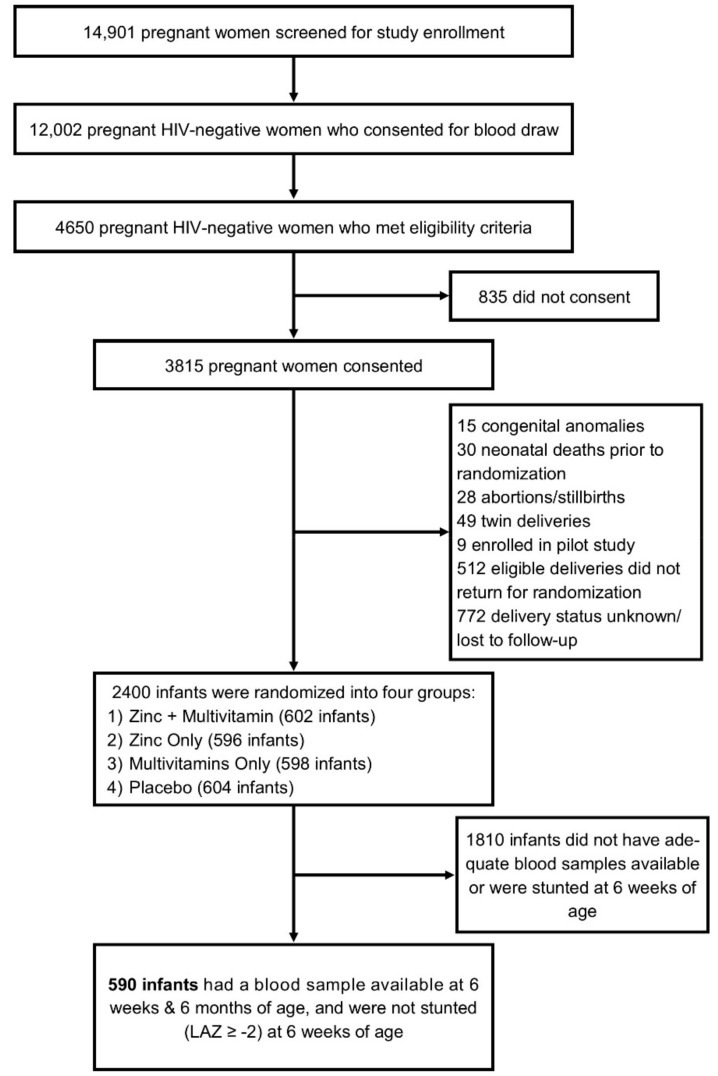
Flowchart demonstrating the selection criteria for our patient population.

For the current post hoc analysis, a subset of children were chosen if they: (1) had blood samples available at 6 weeks and 6 months of age, and (2) were not stunted (LAZ ≥ −2) at 6 weeks of age (Figure 1).

### 2.2. Biological Specimen Collection and Plasma Biomarker Measurement

Blood samples were obtained from children at baseline, 6 weeks, and 6 months of age. Samples were centrifuged and plasma removed within 2 h of blood collection; aliquots were stored in –80 °C freezers until shipped on dry ice for analysis. Serum samples were tested for biomarkers of growth (IGF-1, IGFBP3) and systemic inflammation (AGP, CRP). IGF-1, IGFBP-3, CRP, and AGP were measured by an ELISA method from R&D Systems (Minneapolis, MN, USA).

### 2.3. Statistical Analyses

Data were double entered using Microsoft Access software (Microsoft Corporation, Redmond, WA, USA) and then converted to SAS datasets and uploaded to a secured Unix-based server for analysis. Descriptive statistics were used to summarize baseline characteristics of the study population. FrequDencies were reported for categorical variables and the mean ± standard deviation (SD) for continuous variables. The concentrations of IGF-1 and IGF-BP3 at 6 weeks and 6 months of age were categorized into quartiles and then used to examine their association with child growth. CRP concentrations increase quickly in response to acute insult, peaking at approximately 48 h and decreasing within a week, with a half-life of 19 h [19]. In contrast, AGP concentrations increased more slowly and remained elevated for a longer period of time [20]. Taken together, CRP and AGP measurements can be used to classify individuals who have inflammation from incubation to early convalescence and into late convalescence [21]. Therefore, we explored the association of inflammation with growth by defining systemic inflammation as a composite variable of either CRP > 5 mg/L [22] or AGP > 1 g/L [23] and also by categorizing each CRP and AGP individually as follows: CRP > 5 mg/L [22] or AGP > 1 g/L [23].

Cox proportional hazard models were constructed to estimate hazard ratios (HRs) and corresponding 95% confidence intervals (CIs) for time to stunting, wasting, and underweight across the quartile category of each biomarker and the presence of any inflammation. *p*-value for trend was calculated by including the median value within each quartile as a continuous term in the regression model. Each growth outcome was modeled separately, and the first time the child reached a score of <−2 SD signified an “event.” Children who did not develop an “event” were censored from the study at the time of last anthropometric assessment. In multivariate analysis, we further adjusted for potential confounders including child sex, preterm birth, maternal age, maternal mid-upper arm circumference (MUAC), maternal education, number of household assets (from a list that included sofa, television, radio, refrigerator, and fan), treatment arm of parent trial, anthropometric *z-*score at baseline, diarrhea, malaria, unscheduled clinical visits, or hospitalization. These covariates were selected based on a *p* value < 0.10 in univariate analysis or traditionally considered as a risk factor for child growth outcomes in the literature or biological pathway plausibility. We adjusted for morbidity variables (e.g., diarrhea, malaria, unscheduled clinical visits, hospitalization) in the analysis because we were aware of the possibility that the relationship between inflammation and poor growth may be confounded by the high prevalence of infectious morbidities, which we previously reported in our cohort [24]. Missing data were retained with use of the missing indicator method [25]. *p*-values were 2-sided, with *p* < 0.05 considered statistically significant. All analyses were performed using SAS version 9.4 (SAS Institute, Cary, NC, USA).

### 2.4. Ethics Statement and Study Data

Institutional approval was granted by the Harvard T.H. Chan School of Public Health Human Subjects Committee (HSPH IRB 12875-129), the Muhimbili University of Health and Allied Science Committee of Senate Research and Publications, the National Institute of Medical Research of Tanzania, and the Tanzania Food and Drug Authority, and the parent trial was registered at clinicaltrials.gov as NCT00421668. All mothers provided written informed consent for the parent trial and subsequent analyses.

## 3. Results

Of the 2400 children who were enrolled in the parent trial for multivitamin and/or zinc supplementation between August 2007 and December 2009, 590 met the eligibility criteria and were included in the current substudy. Table 1 summarizes the baseline characteristics of the mothers and children in our cohort. Average maternal age was 26 years, and almost 75% of mothers had one to seven years of formal education. One-third had only one prior pregnancy, and the majority were married or cohabitating with a partner. About 50% of all infants were female, and 3% were born with low birth weight. Mean ± SD LAZ, WLZ, and WAZ scores at 6 weeks and 6 months of age are reported in Table 1.

The incidences of stunting, underweight, and wasting during the follow-up period were 26%, 20%, and 18%, respectively. We previously reported [24] data regarding infectious morbidities in our cohort. For the purpose of this analysis, 1.4% and 1.2% of children had hospitalizations reported and 18.3% and 14.6% had unscheduled outpatient visits reported after 6 weeks and after 6 months, respectively, until the end of study follow-up.

**Figure 2:**
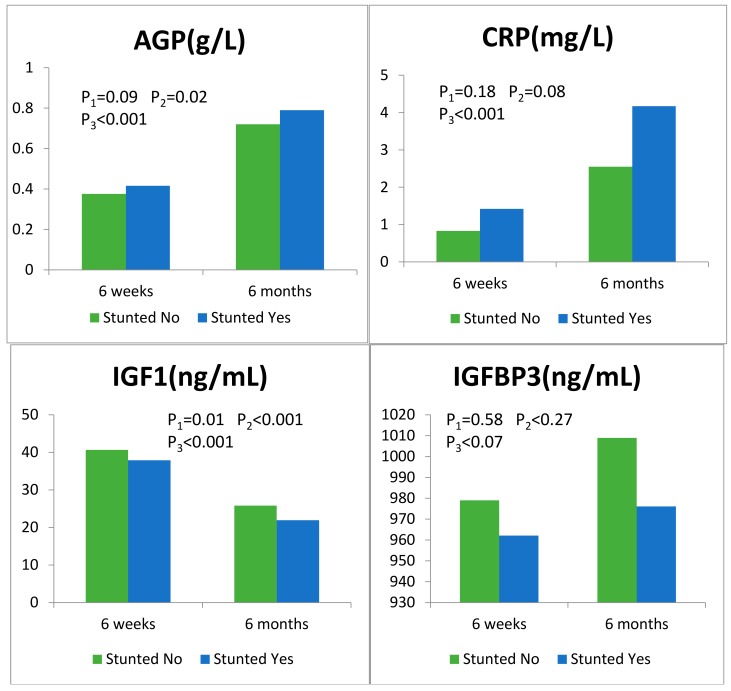
Levels of inflammatory (C-reactive protein (CRP), alpha-1-acid glycoprotein (AGP)) and growth (insulin-like growth factor-1 (IGF-1), insulin-like growth factor binding protein-3 (IGFBP-3)) biomarkers at 6 weeks and 6 months in Tanzanian children who did or did not develop subsequent stunting. Please note: In this figure “stunted” is defined as children who developed stunting during the follow-up period.

All biomarker concentrations had skewed distributions, therefore we report mean ± SE concentrations over the follow-up period. Levels of CRP, AGP, and IGF-BP3 increased significantly and serum concentrations of IGF-1 decreased significantly during the follow-up period, as shown in Figure 2.

Levels of CRP and AGP increased significantly, with mean ± SE (Standard Error) concentrations at 6 weeks and 6 months, respectively, as follows: CRP: 0.99 ± 0.15 mg/L, 3.02 ± 0.33 mg/L, *p* < 0.001; AGP: 0.39 ± 0.01 g/L, 0.74 ± 0.01 g/L, *p* < 0.001. Serum concentrations of IGF-1 significantly (*p* < 0.001) decreased over time: mean ± SE concentration was 39.92 ± 0.49 ng/mL at 6 weeks, 24.78 ± 0.42 ng/mL at 6 months. Likewise, mean ± SE IGFBP3 significantly (*p* < 0.001) increased from 976.4 ± 13.5 ng/mL at 6 weeks to 1000.37 ± 13.11 ng/mL at 6 months. *p*_1_, comparing biomarker between stunted status (yes/no) at 6 weeks; *p*_2_, comparing biomarker between stunted status (yes/no) at 6 months; *p*_3_, comparing biomarker beween 6 weeks and 6 months.

Table 2 summarizes the Spearman’s correlation coefficients relating biomarkers of growth metabolism with inflammation. Our findings were notable for a strong negative association of CRP with IGF-1 at 6 weeks and at 6 months. AGP had a strong negative association with IGF-1 but only at 6 months. AGP and CRP were strongly and positively associated with each other at both time points. Six-week levels of AGP, IGF-1, and IGFBP3 were positively correlated with their 6 month concentrations.

In multivariate analyses (Table 3), children with evidence of systemic inflammation (CRP > 5 mg/L or AGP > 1 g/L) at 6 weeks were 2.14 times (95% CI: 1.23, 3.72; *p* = 0.002) more likely to become stunted over the course of follow-up than children without these elevated markers of inflammation (both CRP ≤ 5 mg/L and AGP ≤ 1 g/L). Systemic inflammation was also significantly associated with stunting at 6 months of age (Table 4) in the univariate analysis with 1.78 times (95% CI: 1.16, 2.72; *p* = 0.01) increased risk of stunting, but there was no statistically significant relationship in multivariate models (HR 1.14; 95% CI 0.68, 1.93; *p* = 0.62). Children with serum IGF-1 in the highest quartile at 6 weeks and 6 months of age had a 42% (HR 0.58, 95% CI 0.37, 0.93; *p* for trend = 0.019) and 45% (HR 0.55, 95% CI 0.22, 1.12; *p* for trend = 0.07) reduced risk of becoming stunted than children with concentrations in the lowest quartile.

Similarly, in adjusted analyses with underweight as the primary outcome, children with systemic inflammation (CRP > 5 mg/L or AGP > 1 g/L) at 6 weeks showed a trend toward increased risk of underweight (HR: 1.65; 95% CI: 0.80, 3.39; *p* = 0.058) as compared with children without inflammation. In multivariate models, children with IGF-1 in the highest quartile at 6 weeks had 45% (HR 0.55, 95% CI: 0.31, 0.98; *p* for trend = 0.052) reduced risk of becoming underweight than children with concentrations in the lowest quartile. This association was also noted in the 6 month univariate models (HR 0.27, 95% CI: 0.13, 0.55; *p* for trend = 0.002), but this relationship was no longer significant in the multivariable model. Children with IGFBP3 in the highest quartile at 6 months only had 77% (HR 0.23, 95% CI: 0.08, 0.63; *p* for trend = 0.01) reduced risk of becoming underweight than children with concentrations in the lowest quartile in the adjusted model.

We found no significant relationship between any 6 week biomarker with wasting in unadjusted or adjusted models. In the 6 month unadjusted model, children with IGFBP3 levels in the highest quartile had a 58% (HR = 0.42; 95% CI: 0.18, 0.97; *p* for trend = 0.04) lower risk of becoming wasted compared to children with concentrations in the lowest quartile, and this relationship remained significant in the multivariable model (HR = 0.33; 95% CI: 0.11, 1.00; *p* for trend = 0.049). The other biomarkers (CRP, AGP, and IGF-1) were not significantly related to the risk of wasting in 6 month unadjusted and adjusted hazard models. Lastly, as shown in Table 5, in models studying the association of each variable CRP > 5 mg/L and AGP > 1 g/L separately at 6 weeks and 6 months of age with subsequent stunting, wasting, and underweight, only AGP > 1 at 6 weeks was found to be significantly associated with stunting (HR 3.20, 95% CI: 1.38, 7.40, *p* for trend = 0.01).

## 4. Discussion

In this large prospective cohort study using data collected from age six weeks for 18 months, we found that systemic inflammation (CRP > 5 mg/L or AGP > 1 g/L) at 6 weeks of age was significantly associated with increased risk of subsequent stunting. Children with higher levels of IGF-1 at 6 weeks and 6 months were substantially less likely to become stunted or underweight. Finally, our findings were notable for a strong negative association between CRP and IGF-1 at both 6 weeks and 6 months, with 6 week AGP levels strongly and positively associated with subsequent AGP levels at 6 months.

Prior studies reported mixed associations between inflammation and growth factors with subsequent growth faltering. Prendergast et al. [14] performed a case control study of 202 Zimbabwean children and measured biomarkers of inflammation (CRP, AGP, IL-6) and growth hormone-IGF axis (IGF-1, IGFBP3) in infant plasma at 6 weeks and 3, 6, 12, and 18 months. They showed that from 6 weeks to 12 months of age, levels of CRP and AGP were consistently higher and of IGF-1 and IGFBP3 were lower in cases (defined as children with HAZ < −2 at 18 months) versus controls (defined as children with HAZ > −0.5 at 18 months). IGF-1 correlated inversely with inflammatory markers at all time points. In multivariate analysis, higher log_10_ levels of CRP (adjusted OR 3.06 (95% CI 1.34, 6.99); *p* = 0.008) during infancy were significantly associated with stunting. Similarly, we also noted a strong negative correlation of CRP with IGF-1 and showed that early inflammation at 6 weeks as measured by both AGP and CRP was associated with an increased risk of stunting. Our results confirm and extend the Zimbabwean data in several ways. First, we studied a combined inflammation variable of CRP > 5 mg/L and AGP > 1 g/dL, which allowed us to capture infants with evidence of biochemical evidence of either acute or chronic inflammation. Second, our prospective study design allowed us to better illustrate the risk of subsequent growth faltering versus the Zimbabwean nested case-control study. Finally, we collected monthly repeated anthropometric data versus the 3 month measures in Zimbabwe.

Our results are consistent with the hypothesis that inflammation suppresses the growth hormone–IGF axis and demonstrate that children with higher levels of IGF-1 in the first year of life are less likely to become stunted or underweight. This is supported by data from a study in Brazil [13] in which, after adjusting for inflammation (as measured by hsCRP), IGF-1 and IGFBP-3 were positively and significantly associated with height-for-age, weight-for-age, and weight-for-height *z-*scores. The authors also reported a similar negative association of hsCRP with IGF-1 and IGFBP-3. However, contrary to our findings, this study did not find a relationship between hsCRP and anthropometric measures. This study was limited by the use of cross-sectional measures of height compared to our design, in which successive assessments of growth over time were made. Furthermore, they defined malnutrition as children with WAZ scores < −2 and matched “non-malnourished controls” as having WAZ scores > −1, whereas our and others’ use of HAZ better reflects chronic malnutrition [26,27].

Arndt et al. reported associations between fecal markers of inflammation, namely neopterin (NEO), myeloperoxidase (MPO), and alpha-1-antitrypsin (AAT), and short-term linear growth in a birth cohort of 246 Bangladeshi children [15]. They reported that elevated MPO levels, but not NEO or AAT levels, were associated with lower short-term linear growth during the second year of life. Their study, however, did not report whether these changes were reflected in measures of systemic inflammation. In a case-control study, Guerrant et al. measured biomarkers of both systemic (hsCRP, serum amyloid A (SAA), soluble cluster of differentiation 14, lipopolysaccharide binding protein) and enteric (fecal MPO, NEO) inflammation in children from several impoverished communities in Fortaleza, Brazil [16]. They reported that lower SAA levels correlated with stunting at enrollment, and that subsequent mean HAZ scores were significantly lower in those with higher fecal MPO or A1AT (alpha-1 antitrypsin). They also reported a significant correlation between impaired linear growth (delta HAZ) (*r* = −0.132, *p* = 0.046, *n* = 231) and higher values of plasma SAA, but no significant correlations were noted with hsCRP levels and HAZ or WAZ at study start or with delta HAZ. This study, however, enrolled children who were older (14.3 ± 5.4 months) at baseline and had biomarkers measured within 1 month of enrollment with follow-up anthropometry at 2–6 months. In contrast, we enrolled children at 6 weeks of age and collected anthropometric data monthly over the 18-month period of follow-up.

Several strengths of our study merit mention. We followed a large cohort of initially well-nourished children at risk for growth faltering. Our monthly repeated measures of length and weight allowed our analysis to take into consideration that normal human growth is variable, with intervals of rapid growth separated by periods of no measurable growth [28]. We measured serum biomarkers at two time points in the first year of life, in early infancy (6 weeks of age) and at 6 months of age. Finally, we included multiple potentially confounding factors in our multivariable models, including clinically apparent infections. We were limited by the lack of maternal levels of growth/insulin metabolism and inflammatory biomarkers. We were additionally limited by the consideration that several of the micronutrients under study in the parent trial, such as zinc, are of crucial importance for immune response (although treatment arm was considered in all multivariable models).

In summary, we demonstrated that systemic inflammation occurs in Tanzanian infants as early as 6 weeks of age and that this inflammation is strongly associated with subsequent stunting in childhood. In addition, children who had higher levels of IGF-1 at both 6 weeks and 6 months were significantly less likely to become stunted. Our work extends the current literature by defining an early critical period of life when both growth faltering and systemic inflammation occur. The finding that serum measures of inflammation and anabolic growth are significant biomarkers in early infancy for subsequent growth faltering brings up the possibility that subclinical infectious or inflammatory processes, such as have been hypothesized to occur in environmental enteric dysfunction, mediate growth failure in children. These findings provide support for continued focus and the need for intervention as early as the first few months of life in children at risk for stunting who live in resource-constrained regions of the world.

## Figures and Tables

**Table 1:** Baseline maternal and child characteristics, *n* = 590.

Maternal Characteristics	Mean ± SD or *n* (%)
Age, years	26.4 ± 5.0
Formal education, years	
0	11 (1.9)
1–7	434 (73.9)
≥8	142 (24.2)
Employment	
Housewife without income	359 (61.6)
Housewife with income	183 (31.4)
Other	41 (7.0)
Married or cohabitating with partner	526 (89.9)
Prior pregnancies	
None	196 (33.4)
1–4	380 (64.7)
≥5	11 (1.9)
Mid-upper arm circumference (cm)	26.8 ± 3.1
**Household Socioeconomic Characteristics**	
Daily food expenditure per person in household <1000 Tanzanian shillings ^1^	144 (26.0)
Household possessions, ^2^ *n*	
0	178 (30.4)
1–3	339 (57.9)
≥3	69 (11.8)
**Child Baseline Characteristics**	
Male	283 (48.0)
Low birth weight < 2500 g	16 (2.7)
Born preterm < 37 weeks	59 (10.8)
Apgar score ≤ 7 at 5 min after birth	8 (1.5)
**Anthropometrics at 6 Weeks**	
Length-for-age *z*-score	−0.14 ± 1.02
Weight-for-length *z*-score	−0.05 ± 1.18
Weight-for-age *z*-score	−0.20 ± 0.87
**Anthropometrics at 6 Months**	
Length-for-age *z*-score	−0.38 ± 1.13
Weight-for-length *z*-score	0.08 ± 1.17
Weight-for-age *z*-score	−0.24 ± 1.06

Note: ^1^ Approximately USD 0.80 at the time of the study. ^2^ From a list that included sofa, television, radio, refrigerator, and fan. SD, standard deviation.

**Table 2:** Correlation coefficient matrix of inflammatory and growth biomarkers.

Biomarkers *r*_s_	At 6 Weeks				At 6 Months			
	AGP, g/L	CRP, mg/L	IGF-1, ng/mL	IGFBP3, ng/mL	AGP, mg/dL	CRP, mg/L	IGF-1, ng/mL	IGFBP3, ng/mL
**At 6 weeks**								
**AGP, g/L**	−	+0.36 **	−0.16	+0.05	+0.25 **	+0.02	−0.02	−0.01
**CRP, mg/L**		−	−0.19 **	−0.05	+0.03	−0.02	−0.01	−0.01
**IGF-1, ng/mL**			--	+0.49 **	+0.01	−0.02	+0.24 **	+0.16 **
**IGFBP3, ng/mL**				−	+0.05	+0.00	+0.07	+0.48 **
**At 6 months**								
**AGP, g/L**					−	+0.44 **	−0.32 **	−0.09
**CRP, mg/L**						−	−0.20 **	−0.12
**IGF-1, ng/mL**							−	+0.42 **
**IGFBP3, ng/mL**								−

Note: Values are Spearman’s rank-order correlation coefficient (*r*_s_). ** *p* < 0.0001. AGP, alpha-1-acid glycoprotein; CRP, C-reactive protein; IGF1, insulin-like growth factor-1; IGFBP3, insulin-like growth factor binding protein-3.

**Table 3:** Association of inflammatory (CRP, AGP) and growth (IGF-1, IGFBP-3) biomarkers at 6 weeks with subsequent stunting, wasting, and underweight.

	Stunting					Underweight					Wasting				
	Events/*n*	Univariate HR (95% CI)	*p* ^1^	Multivariate ^2^ HR (95% CI)	*p* ^1^	Events/*n*	Univariate HR (95% CI)	*p* ^1^	Multivariate ^2^ HR (95% CI)	*p* ^1^	Events/*n*	Univariate HR (95% CI)	*p* ^1^	Multivariate ^2^ HR (95% CI)	*p* ^1^
**CRP > 5mg/L/AGP > 1g/L**															
No	139/542	1.00	0.01	1.00	0.002	95/532	1.00	0.10	1.00	0.058	74/506	1.00	0.20	1.00	0.14
Yes	15/32	1.96 (1.15–3.33)		2.14 (1.23–3.72)		9/31	1.76 (0.89–3.50)		1.65 (0.80–3.39)		7/30	1.66 (0.77–3.61)		1.74 (0.78–3.90)	
**Insulin-like Growth Factor-1, ng/mL**															
Q1	51/145	1.00	0.006	1.00	0.019	33/136	1.00	0.057	1.00	0.052	23/125	1.00	0.13	1.00	0.15
Q2	38/143	0.66 (0.43–1.00)		0.67 (0.43–1.04)		25/144	0.67 (0.40–1.12)		0.67 (0.39–1.14)		20/132	0.77 (0.42–1.40)		0.79 (0.42–1.46)	
Q3	36/146	0.66 (0.42–1.01)		0.63 (0.41–0.99)		25/145	0.71 (0.43–1.20)		0.72 (0.42–1.23)		22/141	0.82 (0.46–1.48)		0.86 (0.47–1.58)	
Q4	30/145	0.53 (0.34–0.83)		0.58 (0.37–0.93)		21/143	0.57 (0.33–0.99)		0.55 (0.31–0.98)		16/143	0.59 (0.31–1.11)		0.59 (0.30–1.15)	
**Insulin-like Growth Factor Binding Protein-3, ng/mL**															
Q1	39/147	1.00	0.94	1.00	0.26	30/144	1.00	0.27	1.00	0.33	23/130	1.00	0.38	1.00	0.57
Q2	42/147	1.05 (0.68–1.62)		0.91 (0.57–1.43)		29/141	0.99 (0.59–1.65)		0.95 (0.56–1.61)		20/136	0.81 (0.45–1.48)		0.83 (0.44–1.54)	
Q3	33/145	0.83 (0.52–1.31)		0.83 (0.51–1.35)		23/143	0.80 (0.47–1.39)		0.79 (0.45–1.40)		19/142	0.75 (0.41–1.39)		0.84 (0.45–1.58)	
Q4	41/145	1.08 (0.70–1.68)		1.17 (0.74–1.85)		22/145	0.76 (0.44–1.32)		0.79 (0.45–1.39)		19/138	0.78 (0.43–1.44)		0.84 (0.45–1.56)	

Note: ^1^ *p*-values for trend for quartile variables were calculated using median value in each quartile category. ^2^ Models adjusted for child sex, preterm birth, maternal age, maternal MUAC, maternal education, number of household assets, treatment arm, *z-*score at 6 months, diarrhea, malaria, unscheduled clinical visits, and hospitalization. AGP, alpha-1-acid glycoprotein; CRP, C-reactive protein, IGF1, insulin-like growth factor-1; IGFBP3, insulin-like growth factor binding protein-3; MUAC, mid-upper arm circumference.

**Table 4:** Association of inflammatory (CRP, AGP) and growth (IGF-1, IGFBP-3) biomarkers at 6 months with subsequent stunting, wasting, and underweight.

	Stunting					Underweight					Wasting				
	Events/*n*	Univariate HR (95% CI)	*p* ^1^	Multivariate ^2^ HR (95% CI)	*p* ^1^	Events/*n*	Univariate HR (95% CI)	*p* ^1^	Multivariate ^2^ HR (95% CI)	*p* ^1^	Events/*n*	Univariate HR (95% CI)	*p* ^1^	Multivariate ^2^ HR (95% CI)	*p* ^1^
**CRP > 5 mg/L/AGP > 1 g/L**															
No	57/374	1.00	0.01	1.000	0.62	57/398	1.00	0.76	1.00	0.66	39/381	1.00	0.86	1.00	0.62
Yes	34/138	1.78 (1.16–2.72)		1.14 (0.68–1.93)		19/141	0.92 (0.55–1.55)		1.15 (0.62–2.13)		15/144	1.06 (0.58–1.91)		1.21 (0.57–2.54)	
**Insulin-like Growth Factor-1, ng/mL**															
Q1	32/123	1.00	0.0001	1.00	0.07	30/128	1.00	0.002	1.00	0.09	18/127	1.00	0.11	1.00	0.97
Q2	26/127	0.74 (0.44–1.24)		1.15 (0.62–2.16)		20/134	0.61 (0.34–1.07)		0.72 (0.37–1.42)		10/130	0.51 (0.24–1.10)		0.17 (0.05–0.64)	
Q3	22/132	0.59 (0.34–1.01)		0.89 (0.47–1.68)		16/139	0.46 (0.25–0.84)		0.53 (0.26–1.08)		17/134	0.85 (0.44–1.65)		1.35 (0.61–3.01)	
Q4	11/132	0.28 (0.12–0.56)		0.50 (0.22–1.12)		10/140	0.27 (0.13–0.55)		0.56 (0.26–1.21)		9/136	0.43 (0.20–0.97)		0.63 (0.23–1.71)	
**Insulin-like Growth Factor Binding Protein-3, ng/mL**															
Q1	26/125	1.00	0.34	1.00	0.45	26/133	1.00	0.009	1.00	0.01	18/131	1.00	0.04	1.00	0.049
Q2	20/134	0.64 (0.36–1.14)		0.90 (0.45–1.80)		21/140	0.73 (0.41–1.30)		0.60 (0.32–1.17)		15/131	0.77 (0.39–1.53)		0.96 (0.40–2.28)	
Q3	27/127	0.99 (0.58–1.70)		1.49 (0.77–2.87)		21/133	0.82 (0.46–1.45)		0.92 (0.48–1.76)		13/135	0.70 (0.34–1.43)		0.75 (0.30–1.91)	
Q4	19/129	0.66 (0.36–1.19)		1.14 (0.55–2.37)		9/136	0.34 (0.16–0.72)		0.23 (0.08–0.63)		8/131	0.42 (0.18–0.97)		0.33 (0.11–1.00)	

Note: ^1^ *p*-values for trend for quartile variables were calculated using median value in each quartile category. ^2^ Models adjusted for child sex, preterm birth, maternal age, maternal MUAC, maternal education, number of household assets, treatment arm, *z*-score at 6 months, diarrhea, malaria, unscheduled clinical visits, and hospitalization. AGP, alpha-1-acid glycoprotein; CRP, C-reactive protein; IGF1, insulin-like growth factor-1; IGFBP3, insulin-like growth factor binding protein-3; MUAC, mid-upper arm circumference.

**Table 5:** Association of CRP and AGP biomarkers at 6 weeks and 6 months with subsequent stunting, wasting, and underweight.

		Stunting					Underweight					Wasting				
		Events/*n*	Univariate HR (95% CI)	*p*	Multivariate ^1^ HR (95% CI)	*p*	Events/*n*	Univariate HR (95% CI)	*p*	Multivariate ^1^ HR (95% CI)	*p*	Events/*n*	Univariate HR (95% CI)	*p*	Multivariate ^1^ HR (95% CI)	*p*
**6 weeks**	**CRP > 5 mg/L**															
	No	135/524	1.00	0.06	1.00	0.07	94/513	1.00	0.54	1.00	0.57	72/488	1.00	0.55	1.00	0.64
	Yes	10/22	1.85(0.97–3.52)		1.86(0.96–3.58)		5/22	1.33(0.54–3.27)		1.30(0.52–3.24)		4/21	1.39(0.51–3.81)		1.37(0.49–3.81)	
	**AGP > 1 g/L**															
	No	145/555	1.00	0.13	1.00	0.01	99/545	1.00	0.19	1.00	0.055	78/518	1.00	0.45	1.00	0.27
	Yes	6/14	1.89(0.84–4.29)		3.20(1.38–7.40)		4/13	1.95(0.72–5.30)		2.75(0.98–7.71)		3/13	1.56(0.49–4.93)		1.97(0.60–6.52)	
**6 months**	**CRP > 5 mg/L**															
	No	76/414	1.00	0.97	1.00	0.55	67/436	1.00	0.19	1.00	0.53	46/424	1.00	0.93	1.00	0.81
	Yes	14/72	1.07(0.61–1.90)		0.82(0.42–1.60)		7/73	0.60(0.27–1.30)		0.76(0.34–1.74)		7/75	0.97(0.46–2.05)		0.89(0.34–2.33)	
	**AGP > 1 g/L**															
	No	62/400	1.00	0.01	1.00	0.76	60/424	1.00	0.98	1.00	0.85	40/408	1.00	0.46	1.00	0.31
	Yes	26/103	1.82(1.15–2.87)		0.92(0.51–1.63)		15/105	0.99(0.56–1.75)		1.07(0.54–2.13)		13/109	1.27(0.68–2.37)		1.50(0.68–3.31)	

Note: ^1^ Adjusted for child sex, preterm birth, maternal age, maternal education, maternal MUAC, number of household assets, treatment arm, baseline *z*-score, and morbidity (hospitalization or unscheduled clinical visits). AGP, alpha-1-acid glycoprotein; CRP, C-reactive protein.
